# Cornea-Specific Human Adipose Stem Cell-Derived Extracellular Matrix for Corneal Stroma Tissue Engineering

**DOI:** 10.1021/acsami.3c17803

**Published:** 2024-03-21

**Authors:** Paula Puistola, Abhinav Kethiri, Antti Nurminen, Johannes Turkki, Karoliina Hopia, Susanna Miettinen, Anni Mörö, Heli Skottman

**Affiliations:** † Eye Regeneration Group, Faculty of Medicine and Health Technology, Tampere University, Tampere 33520, Finland; ‡ Adult Stem Cell Group, Faculty of Medicine and Health Technology, Tampere University, Tampere 33520, Finland; § Tays Research Services, Wellbeing Services County of Pirkanmaa, Tampere University Hospital, 33520 Tampere, Finland

**Keywords:** cell-derived extracellular matrix, cornea-specific, bioink, cornea, human stem cells

## Abstract

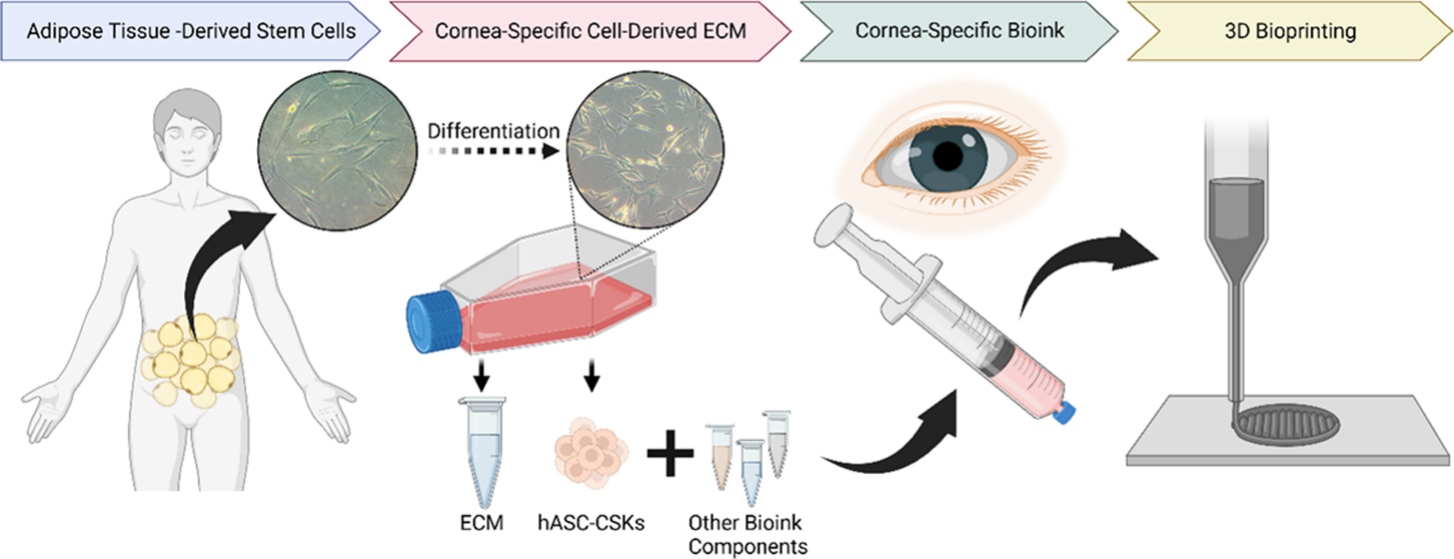

Utilizing tissue-specific extracellular matrices (ECMs) is vital for replicating the composition of native tissues and developing biologically relevant biomaterials. Human- or animal-derived donor tissues and organs are the current gold standard for the source of these ECMs. To overcome the several limitations related to these ECM sources, including the highly limited availability of donor tissues, cell-derived ECM offers an alternative approach for engineering tissue-specific biomaterials, such as bioinks for three-dimensional (3D) bioprinting. 3D bioprinting is a state-of-the-art biofabrication technology that addresses the global need for donor tissues and organs. In fact, there is a vast global demand for human donor corneas that are used for treating corneal blindness, often resulting from damage in the corneal stromal microstructure. Human adipose tissue is one of the most abundant tissues and easy to access, and adipose tissue-derived stem cells (hASCs) are a highly advantageous cell type for tissue engineering. Furthermore, hASCs have already been studied in clinical trials for treating corneal stromal pathologies. In this study, a corneal stroma-specific ECM was engineered without the need for donor corneas by differentiating hASCs toward corneal stromal keratocytes (hASC–CSKs). Furthermore, this ECM was utilized as a component for corneal stroma-specific bioink where hASC–CSKs were printed to produce corneal stroma structures. This cost-effective approach combined with a clinically relevant cell type provides valuable information on developing more sustainable tissue-specific solutions and advances the field of corneal tissue engineering.

## 1 Introduction

The human body is a highly complex system composed of cells, tissues, and importantly extracellular matrix (ECM). Due to the essential role of ECM in providing structural support, biomechanical stimuli, and biochemical signals for the cells,^1^ developing tissue-specific solutions based on ECM holds great potential in the field of tissue engineering (TE) and regenerative medicine. However, ECM is often derived from animal or human donor tissues, and the source of many donor organs and tissues is scarce.^2^ Hence, using ECM derived from cadaveric donor tissue to develop tissue-specific biomaterials is a contradictory solution, and more sustainable solutions are needed. Cell-derived ECM has gained interest as an alternative raw material in developing tissue-specific biomaterials due to its benefits in replicating the complex composition of native tissues while being produced by standardized and prescreened methods. Furthermore, the cell-derived ECM composition can be customized through the choice or alteration of cell types and their exposure to various stimuli, whereas the composition of tissue-derived ECM is set.^3^

The human cornea is one of the tissues where the shortage of donor tissue is severe, with over 12 million global demand for transplants and only one in 70 patients receiving one. These transplants from cadaveric donors are needed for treating corneal blindness which is the third leading cause of blindness in the world.^4^ Collagen-rich corneal stroma comprises almost 90% of the cornea and plays a vital role in the corneal transparency and mechanical strength due to its highly organized microstructure.^5^ This microstructure can be affected by various corneal stromal pathologies, such as keratoconus, which can lead to corneal blindness, necessitating corneal transplant. In addition to their scarcity, the limitations of donor corneas include incomplete nerve regeneration and cellular repopulation as well as potential immunologic reaction.^6^ Consequently, there is a vast need for artificial corneas to address these requirements. Three-dimensional (3D) bioprinting is a state-of-the-art biofabrication technology to manufacture 3D tissue constructs with precise cellular architecture and addresses the substantial need for transplantable tissues and organs.^1,7^ Therefore, it has gained popularity also in fabricating corneal stromal structures.^8−12^ Previously cornea-specific bioinks have been prepared by using ECM from donor tissue, such as bovine^11^ or porcine corneas.^13^ Furthermore, cell-derived ECM for corneal TE has been studied with corneal endothelial cells (CECs) as a culture substrate,^14^ a corneal endothelium graft,^15^ as a model to study cellular dysfunction,^16^ or in applications for corneal injury repair.^17−19^

To avoid using donor corneal tissues, we derived the corneal stroma-specific ECM from human adipose tissue-derived stem cells (hASCs) differentiated toward corneal stromal keratocytes (hASC–CSKs). As a stem cell source, adipose tissue is one of the most abundant tissue types in the human body.^20^ Due to the abundance, easy isolation, and expansion *in vitro*(20) as well as their immunomodulatory properties,^21,22^ hASCs are a popular stem cell type for TE.^23^ In addition, hASCs have been previously shown to differentiate toward CSKs *in vitro*(24,25) and *in vivo*,^26^ and prior studies conducted by others have shown the capability of hASCs to produce ECM *in vitro.*(27,28) Thus, building on the reported advantageous properties of hASCs, we hypothesize that the ECM derived from hASC–CSKs is a highly potential biomaterial for corneal TE.

In this work, we utilized the corneal stroma-specific ECM produced by hASC–CSKs during their differentiation and, thereafter, applied it for 3D bioprinting. Our previously developed hyaluronic acid (HA)-based bioink^29^ was used as the backbone and the decellularized ECM as a bioink component to prepare a corneal stroma-specific bioink. Finally, hASC–CSKs were 3D bioprinted in the corneal stroma-specific bioink to engineer human corneal stroma structures. By producing the ECM with hASC–CSKs, it is possible to avoid the use of donor corneas and combine the advantageous properties of hASCs with the benefits of cell-derived ECM. To the best of our knowledge, the novel approach developed in this study for engineering hASC–CSK-derived ECM and using it as a component for corneal stroma-specific bioink has not been previously explored in the field of corneal 3D bioprinting. Hence, this research contributes to the progress of scientific developments in tissue-specific solutions, especially in situations where the access to donor tissues is limited, and importantly elevates the state of 3D bioprinting of cornea-specific bioinks to a higher level.

## 2 Experimental Section

### 2.1 Cell Culture and Production of *In Vitro* ECM

**Figure 1:**
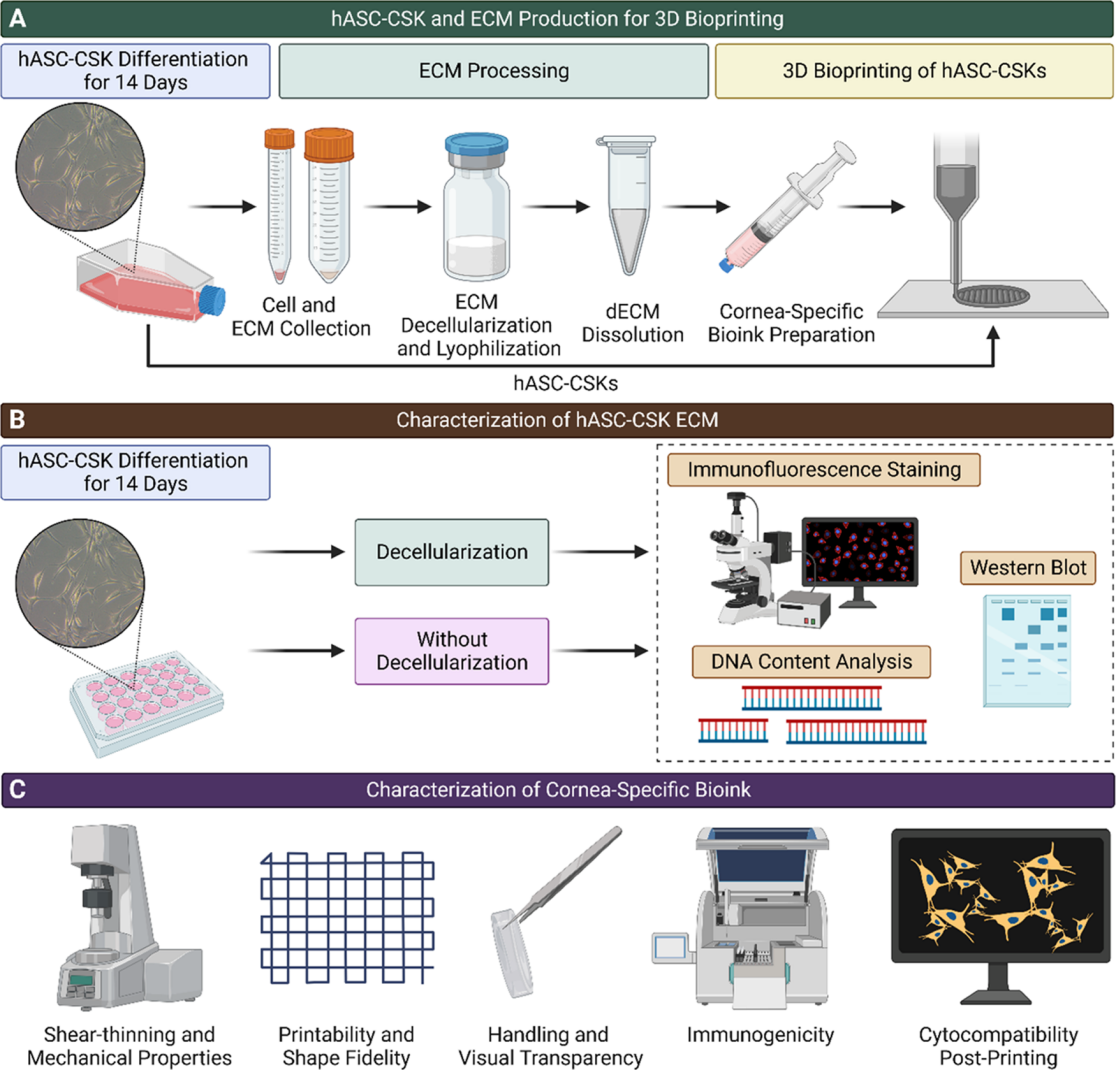
Schematic illustration of the methods. (A) The hASCs were differentiated toward CSKs for 14 days. The produced ECM was collected and utilized as a corneal stroma-specific bioink component used for 3D bioprinting of hASC–CSKs. (B) Characterization of ECM before and after decellularization included IF staining and WB and DNA content analysis. (C) The corneal stroma-specific bioink was characterized by analyzing its shear-thinning and mechanical properties, printability and shape fidelity, handling and visual transparency, as well as immunogenicity and cytocompatibility postprinting.

The production of cells and ECM as well as the ECM processing are summarized in Figure 1A. The isolation and characterization of hASCs from subcutaneous adipose tissue samples was carried out as described previously by Lindroos et al.^30^ Before differentiation, hASCs were expanded in basic culture medium (BM) in T75 flasks (Nunc EasYFlaskTM, Thermo Scientific) at 37 °C with 5% CO_2_. BM was composed of DMEM/F-12 (Gibco) supplemented with 5% human serum (Serana), 1% penicillin/streptomycin (P/S, Gibco), and 1% Glutamax (Thermo Scientific).

The differentiation was started at passage 4 with a seeding density of 7000 cells cm^–2^. For the ECM and cell production, hASCs were seeded in T75 flasks (CellBIND, Corning). For immunofluorescence (IF) characterization of the ECM before and after decellularization, hASCs were seeded on a 24-well plate (CellBIND, Corning). The hASCs were cultured in keratocyte differentiation medium (KDM) for 14 days at 37 °C with 5% CO_2_. KDM was composed of Advanced DMEM (Gibco) supplemented with 1% P/S, 1% Glutamax, 10 ng/mL recombinant human FGF-basic (Peprotech), 0.1 mM l-ascorbic acid 2-phosphate (Sigma-Aldrich), and 1 μM retinoic acid (Sigma-Aldrich). The medium was changed every day for the first 7 days, and thereafter, medium change was done three times a week.

After 14 days, hASC–CSKs were detached from the flasks with TrypLE (Gibco) and used for bioprinting at a density of 2.5 million cells/mL. The ECM sheet produced by the cells was scraped from the flasks and collected in falcon tubes for further processing and characterization. For IF characterization of the ECM, hASC–CSKs on 24-well plates were either fixed as such with 4% paraformaldehyde (PFA) for 15 min at room temperature (RT) or decellularized as described in Section 2.2.

### 2.2 Processing of ECM

Decellularization was performed either on the detached ECM sheets from T75 flasks collected in falcon tubes or directly on hASC–CSKs on 24-well plate without detaching the ECM. First, the cell-containing ECM was incubated in 1% sodium deoxycholate (SD, SAFC, Sigma-Aldrich) prepared in Milli-Q H_2_O for 10 min at RT. Thereafter, ECM was washed with 1× PBS (Dulbecco’s phosphate-buffered saline, DPBS, Carl Roth). To remove any residual DNA, the ECM was treated with DNase I (RNase-free, supplied with MnCl_2_, Thermo Scientific) prepared in MgCl_2_ buffer (Thermo Scientific) and Milli-Q H_2_O at a concentration of 100 U/mL. The DNase treatment was done for 30 min at RT, and thereafter, decellularized ECM was carefully washed with 1× PBS.

After decellularization, the decellularized ECM used as a bioink component was freeze-dried for 24 h. Thereafter, the yield of decellularized ECM was quantified by weighing, and decellularized ECM was stored at −20 °C. Dissolution of decellularized ECM was done in 0.1 M HCl with 0.5 mg/mL pepsin (Roche) at a concentration of 15 mg/mL. The dissolution was carried out under continuous mixing at RT until decellularized ECM was dissolved. Finally, the dissolved decellularized ECM was aliquoted and stored at −20 °C. In addition to processing the collected ECM sheets from the T75 flask to a bioink component after decellularization, some parts of it were flattened out onto a Petri dish for cutting into smaller pieces for IF staining and confocal imaging. The decellularized ECM on the 24-well plate was stained for IF with the decellularized ECM sheet attached to the bottom of the wells.

### 2.3 Characterization of ECM

The ECM characterization methods are summarized in Figure 1B. The decellularization efficacy was determined by performing DNA extraction from ECM (*n* = 15) and decellularized ECM (*n* = 12) with a QIAamp DNA Mini Kit (Qiagen). The DNA extraction was performed according to the manufacturer’s instructions,^31^ and the DNA concentrations were measured with NanoDrop 2000 Spectrophotometer (Thermo Fisher).

The protein composition and corneal stroma specificity of the ECM were analyzed with IF staining and Western Blot (WB). The two-dimensional (2D) IF staining protocol has been previously described by Sorkio et al.^32^ IF staining with keratocan, lumican, collagen types I (Col I) and V (Col V), as well as Hoechst was carried out on decellularized ECM and ECM on 24-well plate as well as detached decellularized ECM sheets from T75 flasks. ECM on a 24-well plate was also stained with phalloidin to illustrate the hASC–CSK morphology before decellularization. The antibody information and dilutions are shown in Table 1. Co-stainings of keratocan with Col I and Col V, as well as co-staining of Col I and V were used to validate the corneal stroma specificity. Stainings of anti-goat A488, anti-rabbit A568, and anti-mouse A568 without primary antibodies were used as secondary antibody controls. After IF staining, the ECM and decellularized ECM on 24-well plate were mounted with antifade mountant (Prolong Gold, Invitrogen) and imaged with a fluorescence microscope (Olympus IX51). The detached decellularized ECM was mounted on glass-bottom dishes (MatTek) with antifade mounting medium (Vectashield, Vector Laboratories) and imaged with a confocal microscope (Zeiss LSM 800).

**Table 1:**
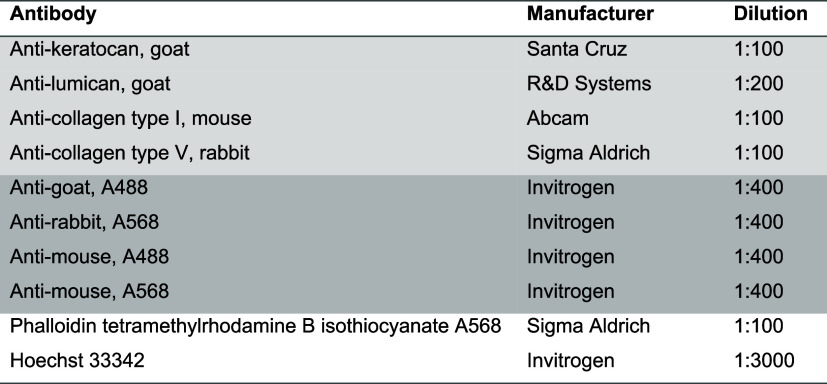
Antibodies, Their Manufacturers, and Dilutions Used in IF Staining of hASC–CSKs^a^

a Primary antibodies are highlighted in light gray and secondary antibodies with darker gray. Phalloidin was used to stain actin filaments of cells, and Hoechst was used to stain cell nuclei.

WB was carried out with Mini-PROTEAN Tetra Vertical Electrophoresis Cell system (Bio-Rad). First, ECM and decellularized ECM were dissolved in 0.1 M HCl with 0.5 mg/mL pepsin and denatured with heating and β-mercaptoethanol treatment. Thereafter, ECM and decellularized ECM were neutralized with 10× PBS and 1 M NaOH, and 50 μg samples were loaded in 4–20% Mini-PROTEAN TGX Precast Protein Gels (Bio-Rad). WB was carried out according to the manufacturer’s instructions^33^ by using anti-Col I (mouse, Abcam, 1:1000), anti-keratocan (rabbit, Santa Cruz, 1:200), and anti-lumican (goat, R&D Systems, 1 μg/mL).

### 2.4 Preparation of Corneal Stroma-Specific Bioink

The bioink backbone was prepared as described previously by Mörö et al.^29^ with slight modifications. The cross-linking components HA-Aldehyde (HA-ALD) and HA modified with dopamine and carbodihydrazide (HA-DA-CDH) were synthesized as described previously by Wang et al.^34^ and Koivusalo et al.,^35^ respectively. Both cross-linking components were dissolved in 1× PBS at a stock concentration of 11 mg/mL. The decellularized ECM and the collagen component of the rheological modifiers were neutralized with 10× PBS (DPBS, Carl Roth) and 1 M NaOH. The two cross-linking components, decellularized ECM, the rheological modifiers, and 1× PBS were mixed in ratios 10:10:5:14:2, respectively. The mixing was done in a dual-syringe system, and the bioink was pre-cross-linked for 50 min at RT before use.

### 2.5 3D Bioprinting Setup

Bioprinting of the corneal stroma-specific bioink was done with an extrusion-based 3D bioprinter 3D-Bioplotter (Manufacturer Series, EnvisionTEC). Bioink was loaded into a 30 cc barrel (Nordson EFD) with a piston and a 30 G blunt needle (0.5″, CELLINK). Perfactory RP software was used to create .stl formats of the 3D models and slice the models with 120 μm intervals. The inner structures of the 3D models were designed with Visual Machines software. The printing was done on 35 mm Petri dishes (TC-treated, Corning). The printing parameters of the corneal stroma-specific bioink were 0.7 bar and 10 mm/s with 0.3 and 0.15 s pre- and postflows, respectively. Two different structures were printed. A 15 × 15 mm^2^ grid with six layers was used for corneal stroma-specific bioink characterization. A cylinder with a 12 mm diameter and 600 μm height was used for evaluating visual transparency as well as bioprinting cells. The distance between filaments was set at 2.5 mm for the grid and 0.35 mm for the cylinder. Grids were printed with one contour and cylinders with two contours with a 0.2 mm distance.

### 2.6 Characterization of Corneal Stroma-Specific Bioink

The characterization methods of the corneal stroma-specific bioink are summarized in Figure 1C. The shear-thinning properties of the bioink were determined by measuring the viscosity with a hybrid rheometer (HR-2 Discovery, TA Instruments). The pre-cross-linked bioink was measured with 20 mm parallel-plate geometry at 20 °C under continuous flow rate with shear rate ranging from 0.01 to 100 s^–1^. A sample size of 500 μL was used with a gap manually set to 1 mm, and four replicates were measured (*n* = 4).

Printability and shape fidelity were determined from the printed grids. The filament thickness and pore factors were analyzed as described previously by Mörö et al.^29^ from six filaments and six pores of the grids immediately after printing and after 7 days in 1× PBS. Four printed grid replicates were analyzed in both time points (*n* = 4).

Mechanical properties of the bioink were determined from the gel disks. The bioink was prepared as described in Section 2.4 and pre-cross-linked in a syringe for 3 h. Thereafter, the pre-cross-linked gel was removed from the syringe and cut into disks with 12 mm diameter. Amplitude and frequency sweeps were measured with an HR-2 Discovery hybrid rheometer after 24 h with 12 mm parallel-plate geometry and a manually set gap of 1.3 mm. Amplitude sweeps were performed with a constant frequency of 1 Hz and oscillation strain ranging from 0.01 to 100%. Frequency sweeps were performed with frequency ranging from 0.1 to 10 Hz and with a constant strain of 10% based on the amplitude sweeps. Three replicates were measured in amplitude sweeps (*n* = 3), and four replicates in frequency sweeps (*n* = 4).

### 2.7 Visual Transparency and Handling of Bioprinted Structures

Transparency after printing was evaluated visually from cylinders printed with and without cells. Transparency of the structures without cells was evaluated immediately after printing and on day 7 postprinting. Transparency with cells was evaluated on day 10 postprinting. Handling was evaluated from cylinders printed without cells on day 7 postprinting. Structures without cells were incubated in 1× PBS, and structures with cells were incubated in KDM at 37 °C with 5% CO_2_.

### 2.8 Immunogenicity of Corneal Stroma-Specific Bioink

The immunogenicity of the bioink without (w/o) and with (w/) decellularized ECM was assessed based on the proliferation of the human peripheral blood mononuclear cells (hPBMCs) on the bioink. Allogeneic hPBMCs were isolated as described by Patrikoski et al.^36^ and cryopreserved in nitrogen gas phase until culture. The rate of the cell proliferation was detected by colorimetric assay using a BrdU ELISA kit (Roche) used according to the manufacturer’s instructions.^37^

The corneal stroma-specific bioink was prepared as described previously in Section 2.4. For the bioink w/o decellularized ECM, the volume of the decellularized ECM component was replaced with 1× PBS. Fibrin sealant (TISSEEL, Baxter) was used as a control material because of its use in clinical applications. In addition, hPBMCs alone were used to determine the baseline response, and culture medium supplemented with 10 μg/mL mitogen phytohemagglutinin (PHA-M, Roche) was used to activate the proliferation of hPBMCs and act as a maximal positive control.

The experiment was carried out on 96-well plates (Nunc MicroWell flat-bottom microplate, Thermo Scientific). Bioinks were mixed and injected into the wells with a sample size of 90 μL and cross-linked for 1 h at 37 °C with 5% CO_2_. Fibrin sealant was loaded into the wells as per the manufacturer’s instructions.^38^ All gels were incubated at 37 °C with 5% CO_2_ in 1× PBS changed twice a day for 4 days before seeding hPBMCs onto the gels. To determine the possible background of the gels, wells containing only the gel without hPBMCs were also prepared. The hPBMCs were cultured in BM or BM supplemented with PHA-M for 4 days, and the medium was changed on day 2. Cells were also imaged on days 2 and 4 to visualize the hPBMC proliferation. After 4 days, hPBMC proliferation was determined with a BrdU ELISA kit and a microplate reader.

The average background values of the biomaterials without cells were subtracted from the average values of the biomaterials with hPBMCs. A total of 11 replicates were used for bioinks w/o decellularized ECM (*n* = 11) and w/decellularized ECM (*n* = 11), 6 replicates were prepared for fibrin sealant (*n* = 6), and 16 replicates for the PHA-M supplemented culture condition (*n* = 16). The value of the hPBMCs alone was used as a baseline proliferation and is indicated as 1. The other conditions were proportioned to the baseline value with values above 1 indicating activated proliferation and, thus, higher immunogenic response.

### 2.9 Cytocompatibility in 3D Bioprinting

The cytocompatibility of the corneal stroma-specific bioink was studied by bioprinting hASC–CSKs in cylindrical 3D structures. The cell viability on days 1 and 7 after printing was evaluated with LIVE/DEAD Viability/Cytotoxicity Kit for mammalian cells (Invitrogen) according to the manufacturer’s instructions.^39^ The samples were imaged with a DMi8 microscope (Leica Microsystems) after 30 min of incubation at 37 °C.

IF staining was used to analyze the cell morphology and cellular interactions after bioprinting on day 10. The 3D IF protocol for bioprinted samples was carried out as described previously by Mörö et al.^29^ Primary antibody anti-connexin 43 1:100 (rabbit, Abcam) was used to stain gap junctions. Anti-rabbit A488 1:400 (Invitrogen) was used as a secondary antibody. Phalloidin tetramethylrhodamine B isothiocyanate A568 (Sigma-Aldrich) 1:80 was used to stain actin filaments of the cytoskeleton. The cell nuclei were stained with Hoechst 33342 at 1:1200 (Invitrogen). After mounting with Vectashield Antifade Mounting medium on MatTek glass-bottom dishes, the samples were imaged with a Zeiss LSM 800 confocal microscope. The z stack images were deconvoluted with Huygens Essential software (Scientific Volume Imaging) and edited in ImageJ Fiji as MIPs. 3D and orthogonal views of the stack images were visualized in Imaris (Oxford Instruments).

### 2.10 Statistical Analysis

The statistical significance of DNA concentration before and after decellularization as well as the shape fidelity analysis of corneal stroma-specific bioink were determined with the Mann–Whitney *U* test, and the immunogenic response of biomaterial conditions with pairwise comparison of Kruskal–Wallis. *P*-values <0.05 were considered statistically significant. SPSS software (IBM) was used for statistical data analysis. All data is presented as mean ± standard deviation.

### 2.11 Ethical Issues

This study was conducted with the approval of Regional Ethics Committee of the Expert Responsibility area of Tampere University Hospital that allows to extraction and use hASCs for research purposes (R15161). Human blood samples, used for buffy coat isolation of hPBMCs, were obtained from the Finnish Red Cross Blood Service, and the study was conducted in accordance with the Declaration of Helsinki 1975, revised in Hong Kong 1989.

## 3 Results

### 3.1 Decellularized ECM Contains Corneal Stroma-Specific Proteins

**Figure 2:**
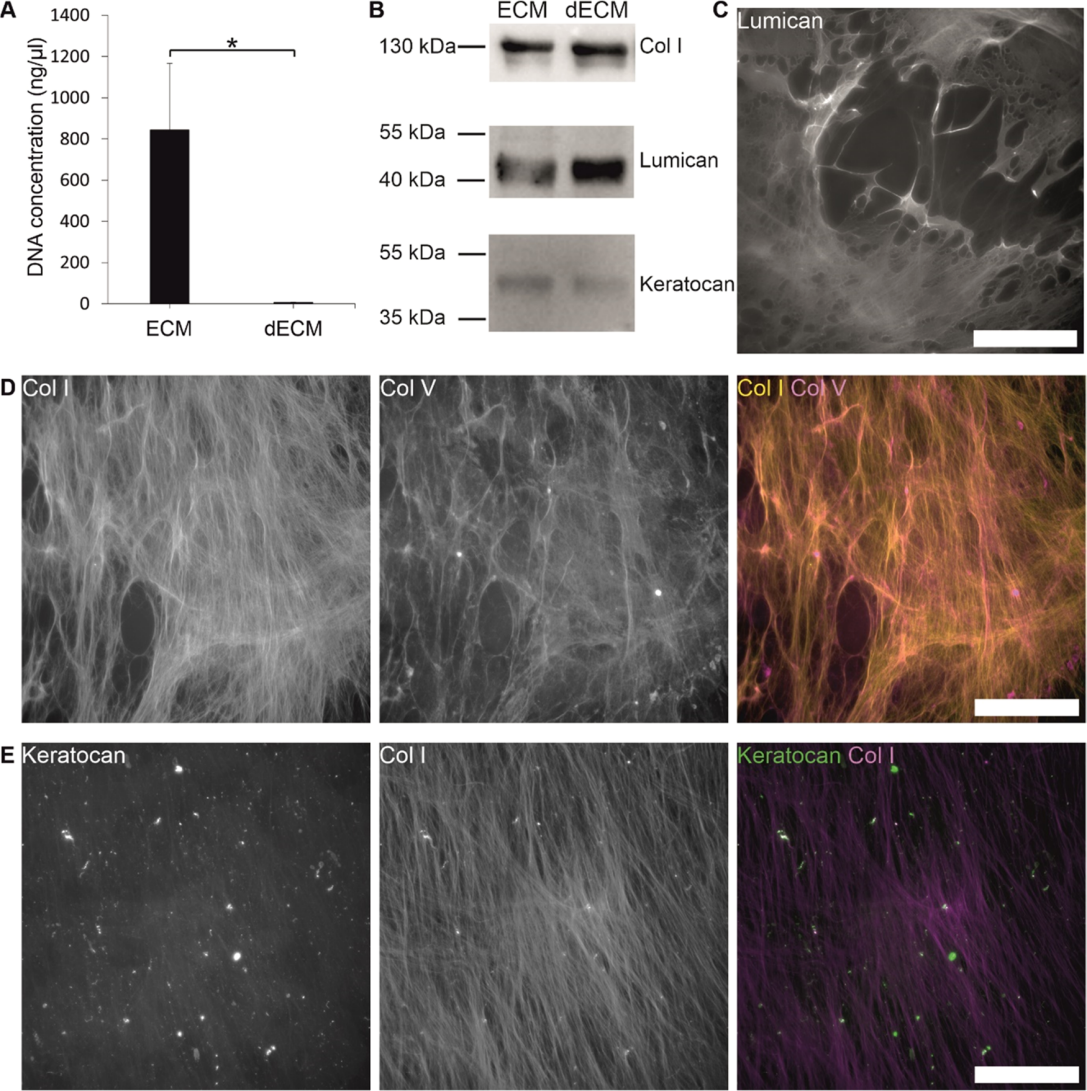
Decellularization efficacy and protein content of the hASC–CSK ECM on day 14. (A) DNA concentration of ECM (*n* = 15) and decellularized ECM (dECM, *n* = 12, *p < 0.05). (B) Western blot analysis of ECM before and after decellularization shows the expression of Col I, lumican, and keratocan. (C) IF staining of lumican (gray). (D) IF staining of collagens type I (yellow) and V (magenta). (E) IF staining of keratocan (green) and Col I (magenta). Scale bars, 200 μm (C–E).

The decellularization efficacy of the hASC–CSK ECM was analyzed by determining the DNA concentration before and after decellularization (Figure 2A). Decellularization was shown to be effective in DNA concentration measurements, and the DNA concentration decreased from 844.0 ± 322.8 ng/μL to 5.8 ± 2.0 ng/μL. This decrease was statistically significant (*p < 0.05). The yield of decellularized ECM after freeze-drying was approximately 10 mg per T75 flask. The protein content of the ECM was characterized by WB and IF staining. The presence of three proteins expressed in native human corneal stroma, Col I, keratocan, and lumican, were detected from both the ECM and decellularized ECM (Figure 2B). The IF staining of proteins was done directly on the wells after the ECM was decellularized. High amounts of lumican (Figure 2C) as well as Col I and V with fiberlike appearance (Figure 2D) were present after decellularization. Extracellular keratocan was present in smaller amounts along Col I fibers (Figure 2D). Thus, hASC–CSKs can produce these ECM proteins which are produced in the native corneal stroma by CSKs. Additionally, the IF staining of the decellularized ECM closely resembled the control IF staining conducted on ECM prior to decellularization (Figure S1), indicating that the decellularization process has no discernible impact on the proteins.

**Figure 3:**
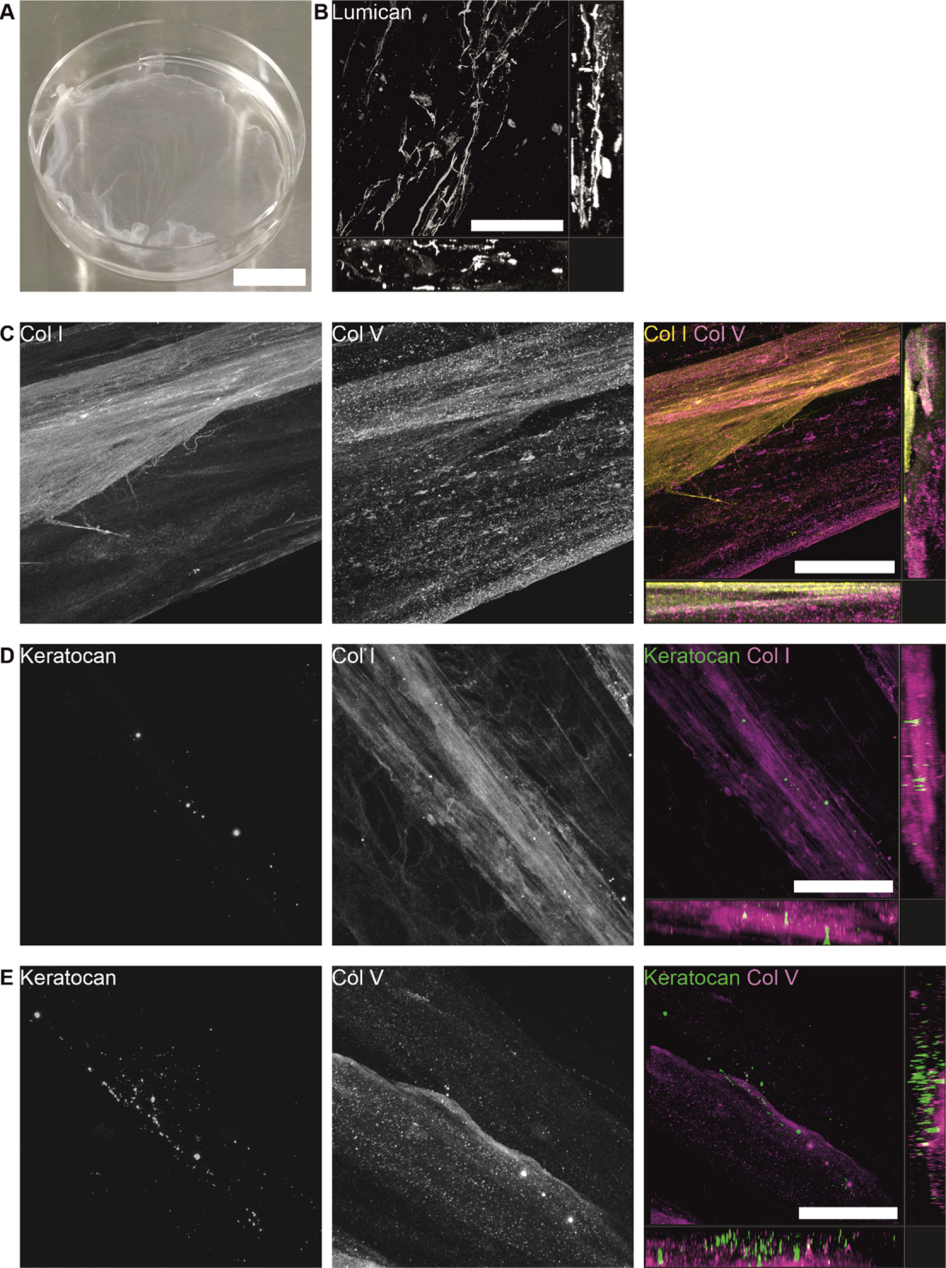
Orthogonal arrangement of proteins in decellularized ECM on day 14. (A) Collected dECM is a transparent sheet and can be transferred to a Petri dish, even after decellularization. Orthogonal visualizations of (B) lumican, (C) Col I and V, as well as keratocan with (D) Col I and (E) Col V. Scale bars: (A) 10 mm and (B–E) 100 μm.

The orthogonal arrangement of proteins in the decellularized ECM sheet was further analyzed by imaging IF stained sheets with a confocal microscope. Even after detaching the ECM sheet from T75 flask for decellularization and spreading the sheet on a Petri dish for IF staining, the decellularized ECM sheet was still intact (Figure 3A). Importantly, its preparation and transfer on glass-bottom dishes for confocal imaging were possible without rupturing the sheet. In orthogonal visualizations, extracellular lumican was seen in fibrillar organization (Figure 3B). Col I and V were visible throughout the cross section of decellularized ECM (Figure 3C), indicating that these collagens are highly expressed in decellularized. Extracellular keratocan was found along the fibers of both Col I and V (Figure 3D,E) with slight local variation in the expression between samples cut from different places of the decellularized ECM sheet.

### 3.2 Corneal Stroma-Specific Bioink Demonstrates Great Printability, Shape Fidelity, and Low Immunogenicity

**Figure 4:**
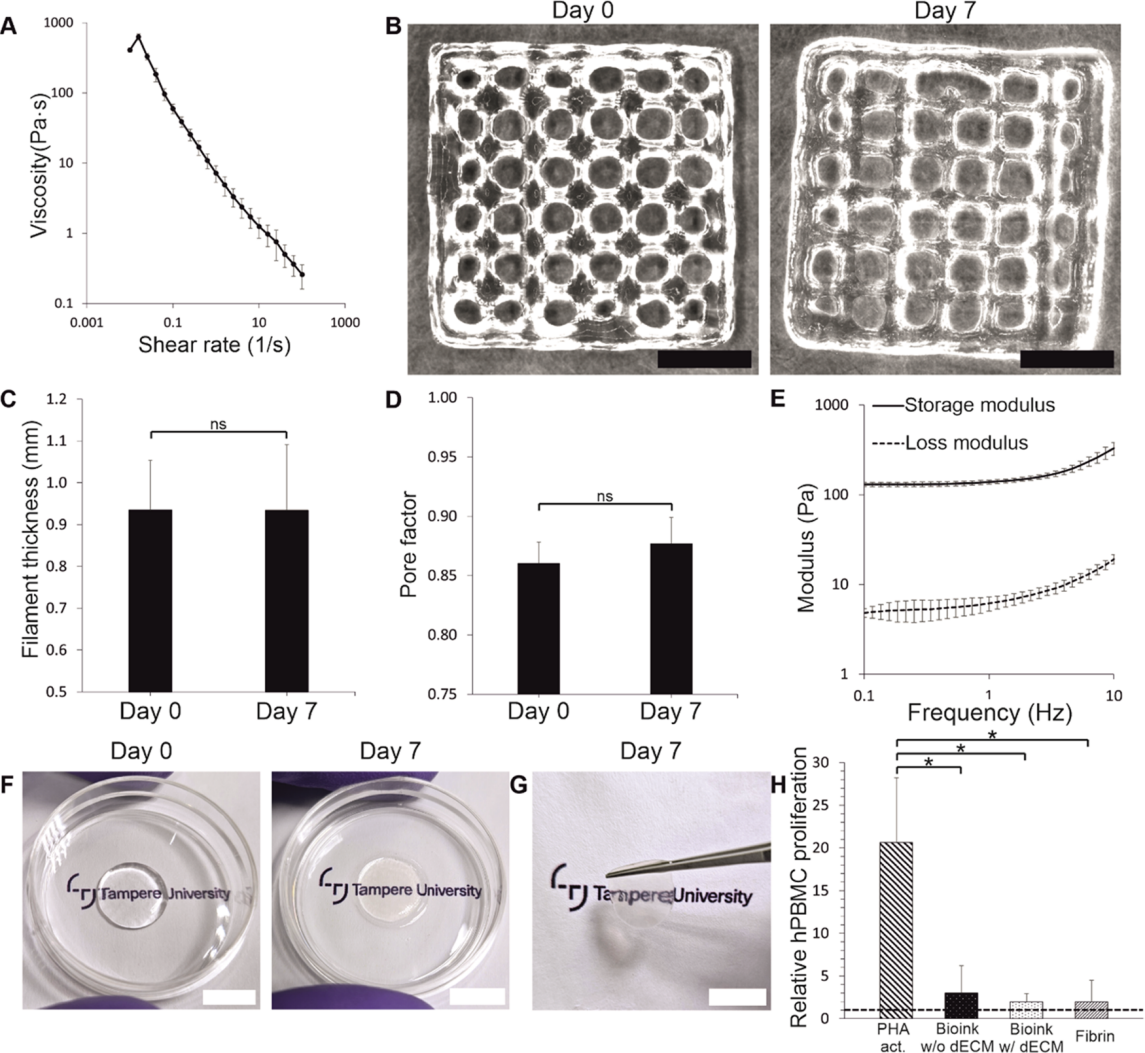
Corneal stroma-specific bioink demonstrates great printability, shape fidelity, and low immunogenicity. (A) The bioink has shear-thinning properties (*n* = 4). (B) The printability and shape fidelity were great when comparing printed grids immediately after printing and after 7 days in PBS. Printability and shape fidelity were further assessed by analyzing (C) filament thickness and (D) pore factor of the grids (*n* = 4). No statistical differences were detected (ns). (E) Mechanical properties of the bioink were analyzed with frequency sweeps (*n* = 4). (F) Transparency of the bioink was assessed from printed structures immediately after printing and after 7 days in PBS. (G) Printed structures were easy to handle after 7 days postprinting. (H) Immunogenicity of bioink w/o and w/decellularized ECM (dECM, *n*_both_ = 11) was compared to fibrin (*n* = 6) by measuring the proliferation of hPBMCs cultured on the biomaterials for 4 days (*p < 0.05). PHA activation was used as a maximum positive control (*n* = 16). The dashed line indicates the baseline response of hPBMCs where values above 1 indicate activation.

The viscosity of the bioink decreased when the shear rate was increased (Figure 4A), and thus the bioink demonstrated shear-thinning properties. The shape fidelity of the bioink was studied by imaging printed grids immediately after printing and after 7 days postprinting (Figure 4B). When analyzing the grids visually, the structure remained intact and attached to the bottom of the printing substrate without showing signs of swelling. The filament thickness was 0.93 ± 0.12 mm on day 0 and 0.93 ± 0.16 mm on day 7 (Figure 4C), and the pore factor was 0.86 ± 0.02 on day 0 and 0.88 ± 0.02 on day 7. Considering that a pore factor closer to 1 indicates a more square than round shape of a pore, the slight increase of the pore factor between days 0 and 7 can be seen visually as more squared pores (Figure 4B). However, no statistically significant differences between time points were detected in filament thickness or pore factor (*p* < 0.05) which indicates great stability and shape fidelity of the bioink.

The viscoelastic properties of the bioink were determined by performing amplitude sweep to determine the linear viscoelastic region (LVR), followed by a frequency sweep at 0.1–10 Hz with a constant strain within the LVR. The measurements were performed on cast gels after 24 h cross-linking (Figure 4E). The average storage and loss moduli at 1 Hz were 138.21 ± 7.26 and 6.15 ± 1.11 Pa, respectively. The storage modulus was higher than the loss modulus throughout the measured frequency range, which indicates that the bioink hydrogel was stable and possessed viscoelastic properties.

The visual transparency of bioprinted structures was analyzed immediately after printing and after 7 days postprinting (Figure 4F). In both time points, text was clearly visible through the bioprinted structure. Furthermore, the size of the structure remained the same, indicating no significant swelling occurred which could have also been seen as decreased visual transparency. The printed structure showed great stability even after 7 days, and it was possible to lift it with tweezers (Figure 4G). Importantly, the structure withstood its own weight while holding it without rupturing or collapsing.

The immunogenicity of the bioink was studied by measuring hPBMC proliferation where the baseline response (hPBMCs alone) was compared to the maximal positive control (PHA activation), bioink w/o and w/decellularized ECM and clinically used fibrin sealant. All of the conditions activated hPBMC proliferation as the detected proliferation was above 1 (Figure 4H). The proliferation was seen in the phase contrast images as well as an increased cell number (Figure S2). The relative hPBMC proliferation of the bioink w/o and w/decellularized ECM as well as fibrin were 3.01 ± 3.19, 1.99 ± 0.94, and 1.98 ± 2.51, respectively. No statistical differences between biomaterial conditions were detected (*p* < 0.05), whereas the maximal positive control provoked a significantly higher response compared to all biomaterial conditions (20.65 ± 7.54, *p < 0.05). The proliferation on the bioink w/o decellularized ECM was slightly higher compared to the other biomaterial conditions, and importantly, the proliferation on the bioink w/decellularized ECM and fibrin sealant was at very similar levels. This indicates the bioink has reasonably low immunogenicity which is important for clinical applications. Interestingly, the bioink w/decellularized ECM condition also had the lowest standard deviation of all conditions, indicating good reproducibility.

### 3.3 Bioprinted hASC–CSKs Have High Viability and Native hCSK-like Morphology in the Corneal Stroma-Specific Bioink

**Figure 5:**
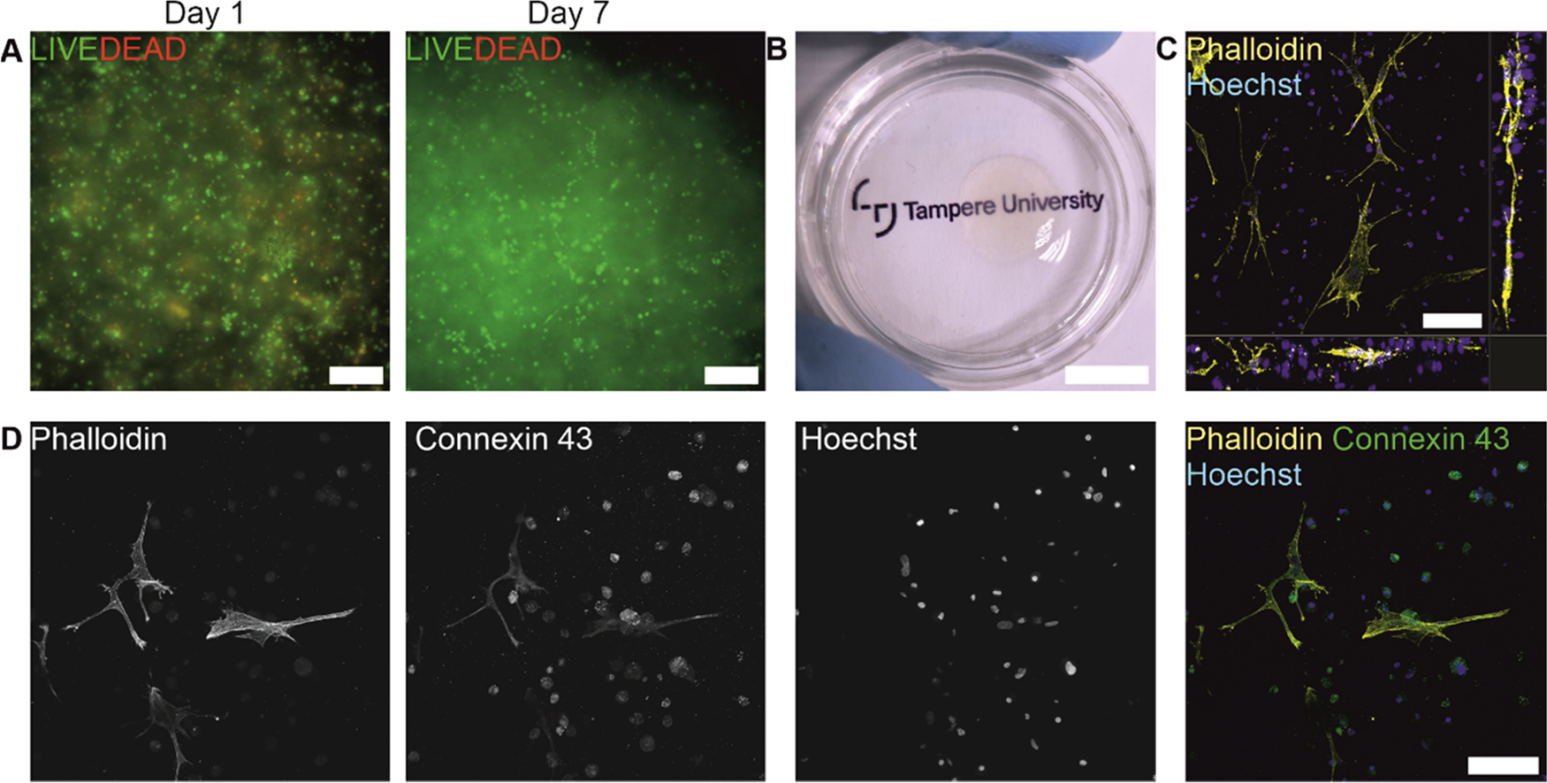
Cytocompatibility of corneal stroma-specific bioink with hASC–CSKs. (A) Postprinting cell viability was good after 1 and 7 days (green = live cells, red = dead cells). (B) Transparency of cell-laden bioprinted structure was sufficient after 10 days postprinting. (C) Orthogonal visualization of the cell morphology after 10 days postprinting. (D) After 10 days, bioprinted hASC–CSKs expressed connexin 43 marker (green), indicating the formation of cell–cell junctions. Cell morphology is illustrated with phalloidin (yellow, C, D) and nuclei with Hoechst (magenta, C, D). Scale bars (A) 200 μm, (B) 10 mm, and (C, D) 100 μm.

The cytocompatibility of corneal stroma-specific bioink was determined by 3D bioprinting hASC–CSKs. The cell viability on days 1 and 7 was good (Figure 5A). The visual transparency of cell-laden structures on day 10 was sufficient for text to be clearly visible through the structure. However, the cells within the bioprinted structure caused some haziness (Figure 5B). The morphology of the bioprinted hASC–CSKs on day 10 was stellate, and cells had formed long extensions (Figure 5C). In the orthogonal visualization, hASC–CSKs can be seen to grow within a printed layer. After 10 days postprinting, hASC–CSKs expressed connexin 43 (Figure 5D) indicating the formation of cell–cell junctions important for tissue formation.

## 4 Discussion

Compared to conventional biofabrication technologies, 3D bioprinting enables automated, customizable, and rapid manufacturing of human corneal stroma structures. For developing tissue-specific bioinks that provide a biologically more relevant approach to replicate the composition of native tissues, ECM is needed. Cell-derived ECM offers a solution to overcome the drawbacks of animal or human donor tissues, such as limited availability of human cadaveric tissues, potential risk of disease transmission, potentially high immunological reaction, low customizability, and batch-to-batch variation of donor tissue.^3^ As prior research on corneal stroma-specific bioinks is dependent on donor corneas,^11,13^ we built on this a new concept of hASC–CSK-derived corneal stroma-specific ECM and applied it to develop a corneal stroma-specific bioink for bioprinting. In addition to abundance, accessibility, and ease to harvest, hASCs are shown to differentiate into multiple lineages,^40^ such as toward hCSKs *in vitro*(24) and *in vivo.*(41) Moreover, they are studied in clinical trials for treating corneal stroma pathologies, such as keratoconus.^42,43^ Undifferentiated hASCs have been reported to produce ECM in large quantities *in vitro.*(27,28) In a previous study, retinoic acid has been shown to promote a keratocyte phenotype in hASCs upon differentiation toward CSKs and to improve cell proliferation and ECM production.^25^ Building on this, here, the presence of ascorbic and retinoic acid in KDM resulted in the production of ECM sheets by hASC–CSKs which could be further processed to a bioink component.

The immunogenicity of decellularized ECM scaffolds can hamper their clinical translation,^44^ and one of the most critical steps is the effective removal of cells from the ECM since incomplete decellularization can induce higher immunogenic response.^45^ Here, we verified the effective decellularization of the *in vitro* engineered ECM by analyzing its DNA concentration before and after decellularization as well as compared the *in vitro* immunogenicity of bioinks with and without decellularized ECM to that of clinically used fibrin. There are several different methods for decellularization which are categorized into physical, chemical, and enzymatic methods.^46^ We carried out chemical decellularization with SD and enzymatic decellularization with DNase, and this combination has also been used for decellularization human corneas.^47^ The drawbacks of chemical and enzymatic decellularization methods include potential damage on the ECM composition, weakened mechanical properties, and the potential effects of residual reagents.^46^ Interestingly, notable changes due to decellularization were not observed in the protein composition of the *in vitro* engineered ECM in the IF or WB analysis. Importantly, the *in vitro* immunogenicity of the bioinks was at a level similar to that of clinically used fibrin. Consequently, the selected decellularization method did not demonstrate previously reported drawbacks with *in vitro* engineered ECM. Our findings can be due to lower cell density and more loose protein composition of the *in vitro* engineered ECM resulting in shorter incubation times compared to decellularization of denser native *ex vivo* tissues, such as overnight DNase incubation of human corneas.^47^ Furthermore, several studies evidence the immunomodulatory properties of mesenchymal stem cells, including hASCs, and their secreted extracellular vesicles^48−51^ as well as cell-derived ECM sheets.^52,53^ Hence, further research on the immunogenicity of our corneal stroma-specific bioink could be carried out to study its potential immunomodulatory properties.

Even though prior research generally confirms the differentiation potential of hASCs toward hCSKs, corneal stroma-specific extracellular proteins need to be present in the hASC–CSK-derived ECM as well. Furthermore, it has been stated that cells need to be capable of synthesizing and secreting corneal-specific ECM to generate corneal tissue.^25^ Here, the corneal stromal specificity of the decellularized ECM was determined with IF staining and WB. The native corneal stroma contains many different types of collagens, such as fibril forming Col I and V, of which Col I is the most predominant one.^54^ We detected high amounts of fibrillar Col I and V in the *in vitro* engineered decellularized ECM. In native corneal stroma, collagen fibrils are tightly packed in layers and small leucine-rich proteoglycans are involved in regulating collagen fibril organization, and thus, the mechanical strength and transparency of the cornea.^54^ The most predominant proteoglycans lumican and keratocan^54^ were detected in our decellularized ECM with both IF and WB analyses. Moreover, keratocan was detected close to Col I and V, which is in line with its role in native corneal stroma. The local keratocan content was observed to vary between separate pieces of the decellularized ECM sheet. High cell densities during hASC–CSK differentiation have been to promote the differentiation capacity and increase the keratocan expression.^24^ Thus, a slight variation in cell density throughout the cell culture flask might have affected the local keratocan content in the produced decellularized ECM sheets.

Prior research in the field of corneal 3D bioprinting demonstrates the potential of using primary hCSKs in bioinks.^8−10,55,56^ However, the use of allogenic primary hCSKs is challenging due to the limited availability of donor tissue as a cell source. Furthermore, 3D bioprinting of functional tissues and organs requires a high number of cells, yet isolating primary hCSKs and achieving high yields in *ex vivo* expansion is difficult.^57^ Instead of primary hCSKs, we bioprinted hASC–CSKs after collecting the ECM. Consequently, there is no need for donor corneas in any step of the 3D bioprinting process, and the production of corneal stroma-specific biomaterial as well as the cells for bioprinting can be carried out concurrently in a controlled environment. The current bottleneck in developing cell-based therapies includes achieving sufficient cell quantity and quality as well as maintaining the production cost-effective and scalable.^58,59^ Combined with the clinical relevance of hASCs in corneal regeneration,^60^ the bioprinting process introduced in this work offers a more straightforward and cost-effective option for manufacturing corneal stroma structures with reduced cost of goods, possibilities for scaling up and lower batch-to-batch variation.

Cells are known to be sensitive to external stresses, and in extrusion-based bioprinting, the shear-thinning properties of the bioink are important for protecting the cells from harmful shear stress.^61^ Here, our novel corneal-stroma-specific bioink demonstrated shear-thinning in viscosity measurements. The viability of hASC–CSKs was good on day 1 postprinting which indicates that the shear-thinning properties provide sufficient protection for the cells during printing. This is supported by our previous study where good viability of bioprinted hASC–CSKs was detected in the HA bioink used as a base in this study.^29^ Furthermore, good viability on day 7 indicates that the corneal stroma-specific bioink provides a suitable environment for the bioprinted hASC–CSKs. After 10 days, bioprinted hASC–CSKs demonstrated native hCSK-like morphology which is reported to be stellate and show more round cell bodies.^62^ Compared to our previous study with bioprinting hASC–CSKs in a bioink without decellularized ECM,^29^ here the cell morphology was more stellate with longer extensions even after shorter culture period postprinting. This can be due to the addition of decellularized ECM into the bioink or a longer differentiation time before printing. To gain an in-depth understanding of the possible mechanisms of decellularized ECM on cell phenotype characteristics, additional research is needed. Moreover, the bioprinted cells express connexin 43 which indicates cellular interactions. Even though the expression is not as high as we have seen previously by Mörö et al.^29^ and there are still cells with rounded morphology after 10 days postprinting, it should be noted that the culture period postprinting in this study was shorter. Furthermore, it is known that the differentiation decreases the proliferation of the stem cells,^63^ and we have previously seen that hASCs differentiated toward CSKs have lower proliferation capacity and growth rate that is typical for CSKs.^29^ In this study, the differentiation before bioprinting was continued 14 days instead of 7 days, which can result in a slower growth rate of the differentiated cells. Importantly, the cell-laden bioprinted structures were intact after 10 days and did not show any signs of shrinkage or collapse due to cell proliferation.

As a final note for potential applications beyond 3D bioprinting, the ECM sheet could be used alone without processing it to a bioink component. In our study, we noted that the ECM sheet exhibited favorable properties for handling, cutting, and transfer, even after rigorous processing such as vortexing. The resilience was particularly noteworthy as cell-derived sheets often lack mechanical strength due to insufficient thickness.^19,64,65^ Interestingly, ECM sheets hold significant promise for TE and regenerative medicine applications, potentially serving as a therapeutic option for corneal injuries during wound healing. While hASC-derived ECM sheets have been explored for skin substitutes in wound healing,^27,28^ the production of ECM sheet during the differentiation of hASCs toward CSKs is a novel approach. It is important to note that further investigations are needed to conduct mechanical characterization as well as assess biocompatibility and *in vivo* biodegradation of the decellularized ECM sheet in future studies.

## 5 Conclusions

This study introduces a novel approach for regenerative medicine through engineering corneal stroma-specific ECM and utilizing it in 3D bioprinting. To the best of our knowledge, this is the first study where hASC–CSK-derived decellularized ECM containing corneal stroma-specific proteins has been applied in 3D bioprinting corneal stroma structures. The approach eliminates the need for donor corneas, making treatments for corneal blindness more accessible and efficient in the future. Furthermore, this research contributes to addressing the current bottleneck of developing cost-effective methods for cell-based therapies and fabricating corneal stromal structures resembling native corneal stroma. Consequently, this research has the potential to revolutionize the fabrication of corneal transplants and improve the lives of countless patients, ushering in a new era of regenerative medicine.

## Supporting Information Available

IF staining of ECM before decellularization (Figure S1); phase contrast images of hPBMC proliferation on different culture conditions (Figure S2) (PDF)
