# Supplementary material for: Cornea-Specific Human Adipose Stem Cell-Derived Extracellular Matrix for Corneal Stroma Tissue Engineering
Source: ACS Appl Mater Interfaces. 2024 Mar 21;16(13):15761–72. doi: 10.1021/acsami.3c17803 (PMC10995904; doi:10.1021/acsami.3c17803)
Supplement: Supplementary file 1 — am3c17803_si_001.pdf [file am3c17803_si_001.pdf]

Supporting Information

CORNEA-SPECIFIC HUMAN ADIPOSE STEM CELL - DERIVED EXTRACELLULAR MATRIX FOR CORNEAL STROMA TISSUE ENGINEERING

Paula Puistola[1], Abhinav Kethiri[1], Antti Nurminen[1], Johannes Turkki[1], Karoliina Hopia[1], Susanna Miettinen[2,3], Anni Mörö[1‡], Heli Skottman[1‡]*

[1] Eye Regeneration Group, Faculty of Medicine and Health Technology, Tampere University, Tampere 33520, Finland.

[2] Adult Stem Cell Group, Faculty of Medicine and Health Technology, Tampere University, Tampere 33520, Finland

[3] Tays Research Services, Wellbeing Services County of Pirkanmaa, Tampere University Hospital, 33520 Tampere, Finland.

‡ Shared last authorship

* Corresponding author: heli.skottman@tuni.fi

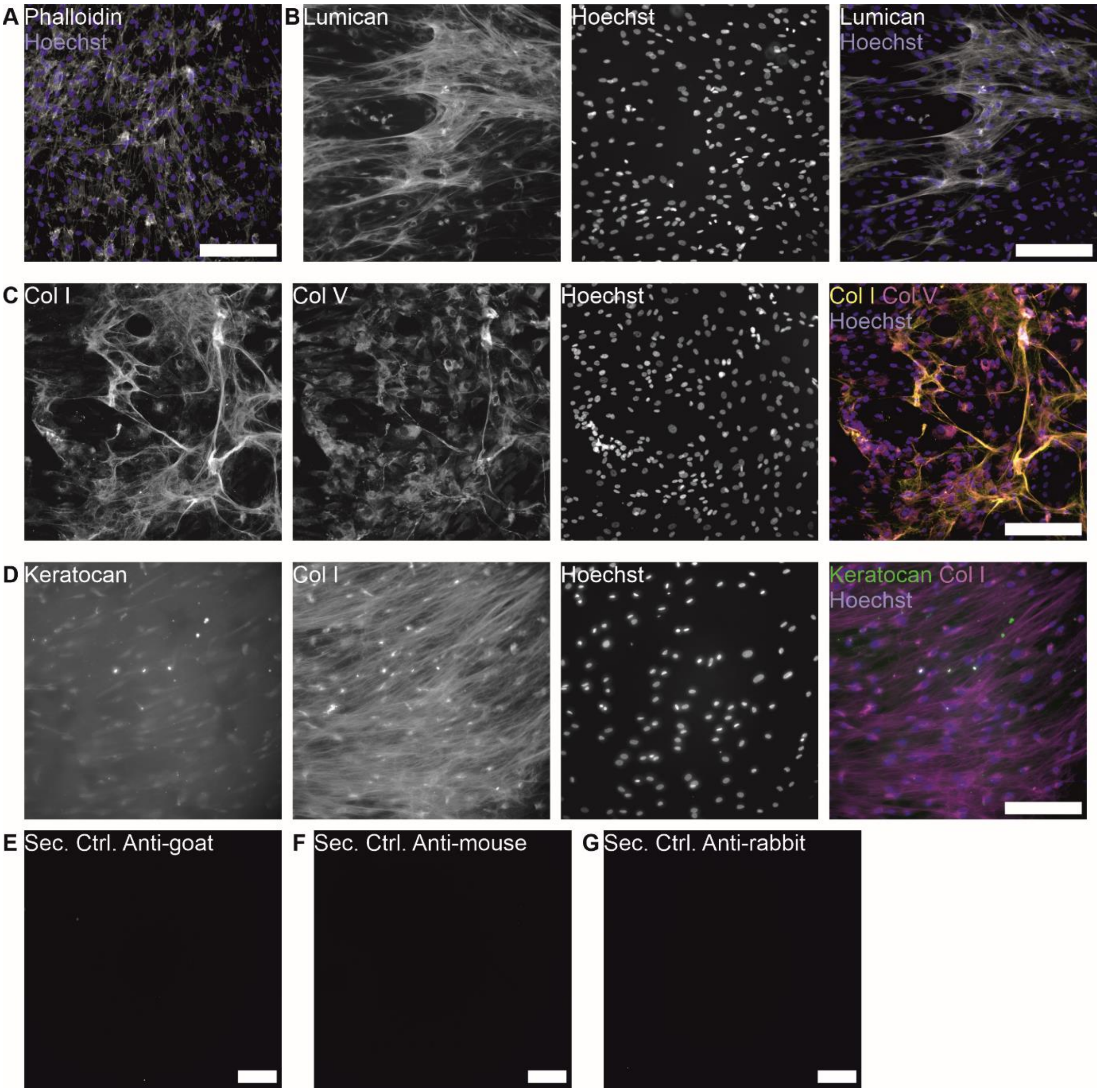

Figure S1. IF staining of hASC-CSK ECM before decellularization. (A) Cell morphology after 14 days illustrated with phalloidin (grey). Day 14 IF staining of (B) lumican (grey), (C) Col I (yellow) and V (magenta), and (D) keratocan (green) along Col I fibers (magenta). Nuclei visualized with Hoechst (blue, A-D). Secondary antibody controls of hASC-CSKs 1/18 at day 7 before decellularization for (E) anti-goat (F) anti-mouse and (G) anti-rabbit. Scalebars 200 µm (A-G).

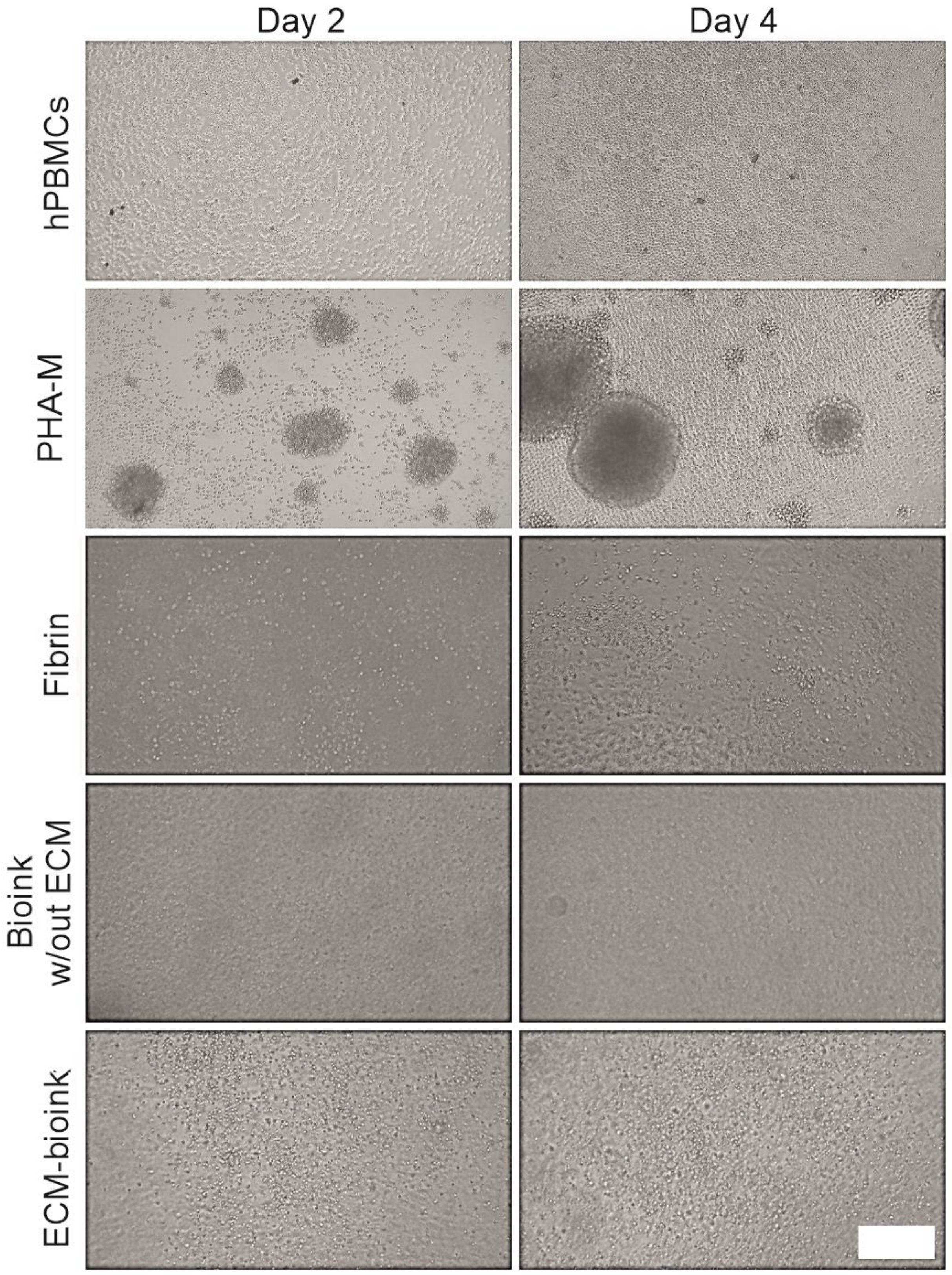

Figure S2. Proliferation of hPBMCs on biomaterials. Phase contrast images showing the proliferation of hPBMCs on day 2 and 4 in different conditions. Higher proliferation at day 4 indicates higher immune response of the condition. Phytohemagglutinin-M (PHA-M) condition shows the highest cell proliferation response with increased cell clusters (maximal positive control). Scale bar 200 µm.
